# The clock gene *PER1* suppresses expression of tumor-related genes in human oral squamous cell carcinoma

**DOI:** 10.18632/oncotarget.7827

**Published:** 2016-03-01

**Authors:** Han-Xue Li, Xiao-Juan Fu, Kai Yang, Dan Chen, Hong Tang, Qin Zhao

**Affiliations:** ^1^ Department of Oral and Maxillofacial Surgery, The First Affiliated Hospital of Chongqing Medical University, Chongqing 400016, China

**Keywords:** oral cancer, tumor, circadian clock, PER1, tumor-related genes

## Abstract

Abnormal expression of the clock gene *PER1* is highly correlated with carcinogenesis and the development of malignant tumors. Here, we designed short hairpin RNAs (shRNAs) to effectively knock down *PER1 in SCC15 human oral squamous cell carcinoma cells. shRNA-mediated PER1* knockdown promoted SCC15 cell growth, proliferation, apoptosis resistance, migration and invasion *in vitro*. *PER1* knockdown also increased the cells' expression of *KI-67, MDM2, BCL-2, MMP2* and *MMP9* mRNA, and decreased expression of *C-MYC, p53, BAX* and *TIMP-2*. In BALB/c nu/nu nude mice subcutaneously injected with SCC15 cells, *PER1* knockdown in the cells enhanced tumor development, leading to increased tumor weights and volumes. These results suggest that *PER1* is an important tumor suppressor gene and may be a useful molecular target for the treatment of cancer.

## INTRODUCTION

Most biological activities in the lives of mammals, such as hormone secretion, cellular functionand metabolism, fluctuate over a 24-h period. This fluctuation, known as circadian rhythm, is generated through circadian variation in the expression of clock genes in nearly every cell in the body [1–6]. Light is one of the factors affecting circadian rhythm, but in the absence of light, the activities of life continue to operate over an approximately 24-h cycle. Thus, the circadian rhythm is one of the basic characteristics of an organism's life [2, 5, 7].

King et al. cloned the first mammal clock gene, *Clock*, in 1997 [8]. Since then, at least 14 core clock genes have been reported, including *PER1, PER2, PER3, CRY1, CRY2, ClOCK, BMAL1, TIM, CK1ε, NPAS2, REV-ERBs, DEC1, DEC2* and *RORs* [2, 3, 7, 9, 10]. Clock genes have three important functions [2, 4, 5]. First, circadian rhythm generated by circadian variation in clock gene expression maintains a high level of coordination and synchronization among different and complicated physiological processes. Second, the internal clock can be reset in response to external changes to better adapt to the environment. Third, clock genes control approximately 2%-10% of the genes in a mammal's genome. These are known as clock-controlled genes (CCGs), and can affect cellular activities by altering expression downstream CCGs [11–13]. Moreover, recent studies have shown that aberrant expression and altered clock gene rhythms are associated with pathogenic conditions, including cancer, obesity and depression [9, 14, 15-17].

*PER1* is an important clock gene that stabilizes the duration of circadian rhythm. Abnormal expression of *PER1* in mammals is not only associated with circadian rhythm disturbances, but is also closely correlated with carcinogenesis and the development of cancers. Because there is a close relationship between the circadian rhythm and the cell cycle, aberrant *PER1* expression can lead to abnormal expression of numerous downstream cell-cycle genes, including *CyclinB1, CyclinD, Cyclin E, WEE-1, CDK1* and *p53* [6, 20, 21]. It has therefore been suggested that *PER1* can inhibit malignant cell transformation by altering the cell cycle and promoting cell-cycle checkpoint repair in response to DNA damage [6, 20]. However, carcinogenesis is a complex process involving cell growth, proliferation, apoptosis, invasion, metastasis and tumor angiogenesis [7, 9, 19, 22-24]. For that reason, in the present study we further investigated the relationship between *PER1* and carcinogenesis. Our findings clarify the tumor suppressor role played by *PER1* during carcinogenesis.

## RESULTS

### Construction of lentivirus shRNA plasmids

DNA sequencing showed the lentivirus PER1-shRNA-I-III plasmids to be exactly the same as the respective sense strands (Supplemental Figure S1 and Supplemental Table S1), which indicates the three shRNAs targeting *PER1* were successfully constructed.

### Levels of PER1 mRNA and protein in tumor cells

**Figure 1:**
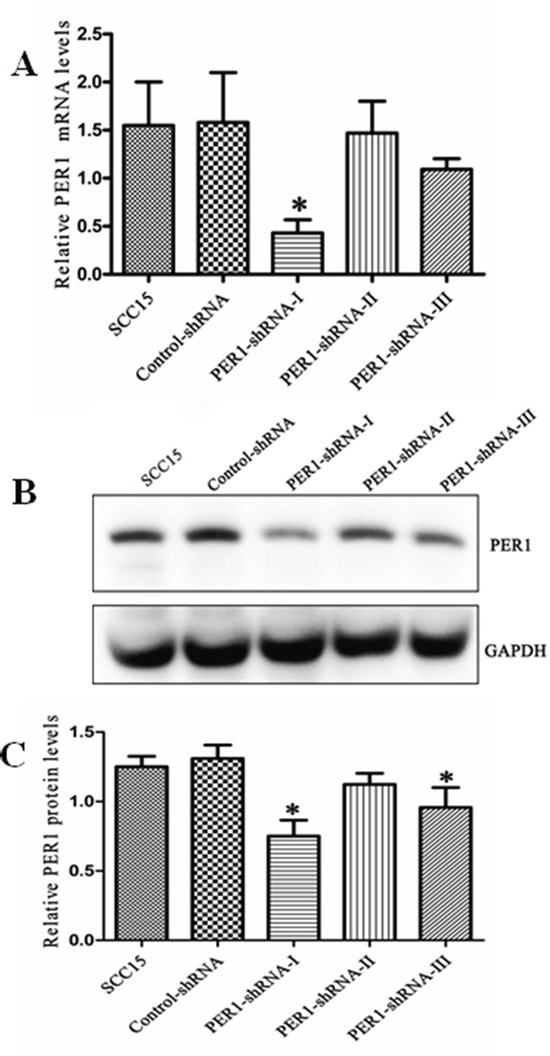
*PER1* is efficiently knocked down in SCC15 cells transfected with PER1-shRNA-I **A.** Levels of *PER1* mRNA were significantly reduced in SCC15 cells transfected with PER1-shRNA-I. **B.** Levels of PER1 protein in the SCC15, Control-shRNA and PER1-shRNA-I-III groups. **C.** Expression of PER1 protein was significantly down-regulated in SCC15 cells transfected with PER1-shRNA-I or PER1-shRNA-III. Data were presented as the mean±SD. Significant differences between multiple groups were evaluated using ANOVA; differences between two groups were evaluated using the LSD test. *P<0.05.

The relative level of PER1 mRNA (protein) normalized to the level of GAPDH mRNA (protein) was 1.58±0.52 (1.25±0.08) in untreated SCC15 cells, 1.55±0.45 (1.31±0.10) in cells expressing Control-shRNA, and 0.43±0.14 (0.75±0.12), 1.47±0.33 (1.12±0.08) and 1.09±0.11 (1.00±0.14), respectively, in cells expressing PER1-shRNA-I, -II or -III (Figure 1A-1C). Thus expression PER1-shRNA-I significantly (*P<0.05*) reduced levels of both PER1 mRNA and protein by about half as compared to the other groups. This demonstrates that PER1-shRNA-I effectively knocked down *PER1* expression, and so it was used for the following experiments.

### Growth and proliferation of tumor cells

**Figure 2:**
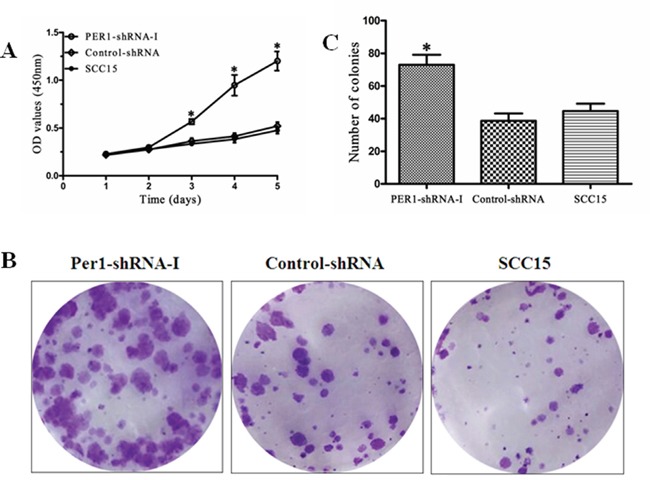
*PER1* inhibits SCC15 cell growth and proliferation **A.** CCK-8 assays of SCC15 cells in the PER1-shRNA-I, Control-shRNA and SCC15 groups. **B.** Representative micrographs of colonies formed by cells in the PER1-shRNA-I, Control-shRNA and SCC15 groups (crystal violet staining). **C.** The colony formation rate was significantly higher in SCC15 cells transfected with PER1-shRNA-I. Data were presented as the mean±SD. Differences between multiple groups were evaluated using ANOVA; differences between two groups were evaluated using the LSD test. *P<0.05.

The results of CCK8 assays are shown in Figure 2A. Cell growth was obviously increased in the PER1-shRNA-I group as compared to the Control-shRNA and SCC15 groups (*P<0.05*), while there was no significant difference between the Control-shRNA and SCC15 groups (*P>0.05*). In addition, the colony formation rate in the PER1-shRNA-I group (73.00±6.08 %) was significantly higher than in the Control-shRNA (38.67±4.51 %) or SCC15 (44.67±4.51 %) group, which did not differ (*P>0.05*) (Figure 2B and 2C). This indicates that *PER1* knockdown enhances cell growth potential.

### Tumor cell apoptosis

**Figure 3:**
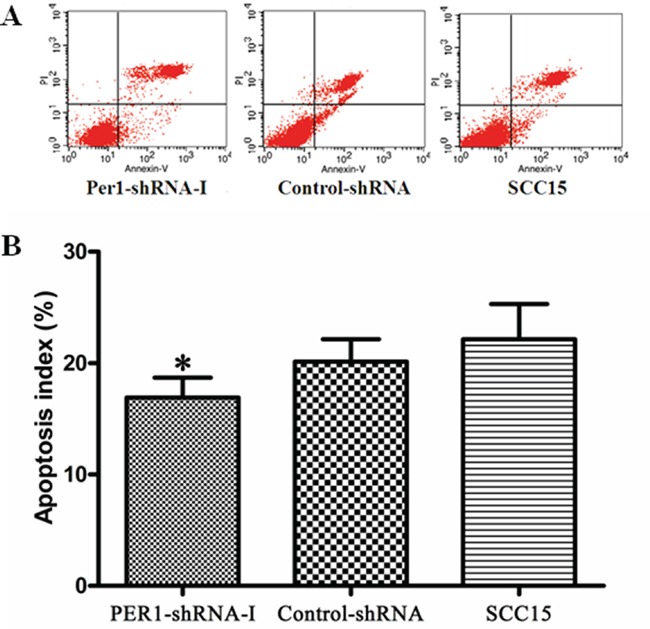
*PER1* promotes SCC15 cell apoptosis **A.** Representative flow cytometry profiles of apoptosis among cells in the PER1-shRNA-I, Control-shRNA and SCC15 groups. **B.** The apoptosis index was significantly reduced in SCC15 cells transfected with PER1-shRNA-I. Data were presented as the mean±SD. Differences between multiple groups were evaluated using ANOVA; differences between two groups were evaluated using the LSD test. *P<0.05.

The cell apoptosis index among cells expressing PER1-shRNA-I (16.91±1.78 %) was significantly lower than among cells expressing Control-shRNA (20.14±2.00 %) or untreated SCC15 cell (22.13±3.17 %), and again no difference was noted between the Control-shRNA and SCC15 groups (Figure 3A and 3B). This indicates that *PER1* knockdown interferes with the progression of apoptosis in SCC15 cells.

### Tumor cell migration and invasion

**Figure 4:**
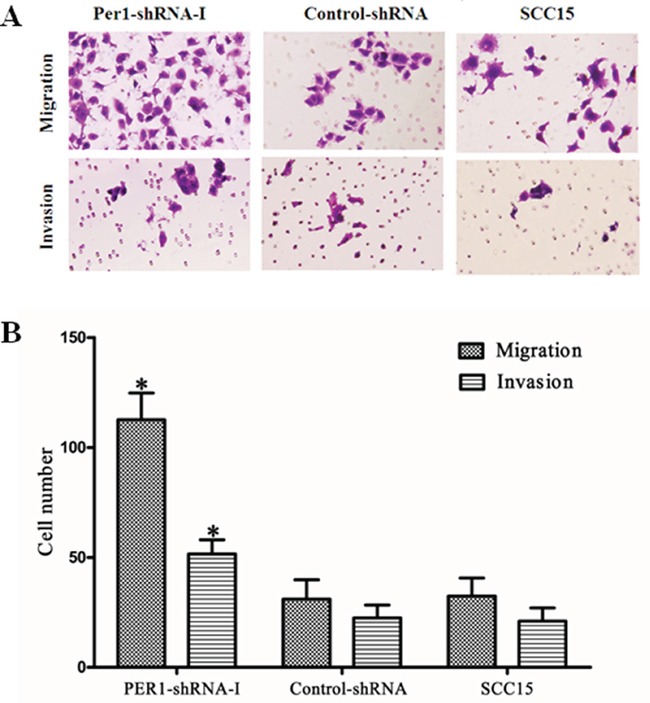
*PER1* suppresses cell migration and invasion by SCC15 cells **A.** Representative micrographs showing migrating and invading SCC15 cells in the indicated groups (crystal violet staining, 200×). **B.** *PER1* knockdown increased migration and invasion by SCC15 cells. Data were presented as the mean±SD. Differences between multiple groups were evaluated using ANOVA; differences between two groups were evaluated using the LSD test. *P<0.05.

In Transwell assays, the average numbers of migrating (invading) cells in the PER1-shRNA-I, Control-shRNA and SCC15 groups were 113±12(52±6), 31±9 (23±6) and 32±8 (21±6), respectively (Figure 4A and 4B). *PER1* knockdown significantly (*P<0.05*) increased both the migration and invasiveness of SCC15 cells as compared to cells in the Control-shRNA and SCC15 groups.

### Levels of mRNA expression of tumor-related genes in tumor cells

Expression of *KI-67, MDM2, BCL-2, MMP2* and *MMP9* mRNA was significantly (*P<0.05*) up-regulated in the Per1-shRNA-I group ascompared to the Control-shRNA and SCC15 groups, while expression of *C-MYC, p53, BAX* and *TIMP-2* mRNA was significantly (*P<0.05*) down-regulated. There was no notable difference between the Control-shRNA and SCC15 groups. In addition, there was no difference in expression of *VEGF* mRNA among the three groups (Table 1).

**Table 1:** Levels of mRNA expression of tumor-related genes in the PER1-shRNA-I, Control-shRNA and SCC15 groups (mean±SD)

Gene	PER1-shRNA-I	Control-shRNA	SCC15	F	P	P_1_	P_2_	P_3_
PER1	0.43±0.14	1.55±0.45	1.58±0.52	7.88	0.021	0.015	0.013	0.931
KI-67	1.25±0.15	0.39±0.11	0.45±0.19	29.18	0.001	0.000	0.001	0.650
MDM2	1.02±0.04	0.58±0.21	0.63±0.20	5.93	0.038	0.020	0.031	0.749
C-MYC	0.69±0.26	1.30±0.27	1.22±0.16	5.98	0.037	0.019	0.032	0.702
p53	1.20±0.23	2.60±0.72	2.78±0.22	10.89	0.010	0.009	0.005	0.643
BAX	0.67±0.08	1.17±0.13	1.04±0.06	22.16	0.002	0.001	0.003	0.164
BCL-2	1.58±0.21	0.87±0.15	0.73±0.14	21.55	0.002	0.002	0.001	0.362
MMP2	1.61±0.19	0.98±0.03	0.95±0.14	23.03	0.002	0.001	0.001	0.749
MMP9	1.63±0.11	0.87±0.15	0.85±0.19	24.67	0.001	0.001	0.001	0.880
TIMP-2	0.82±0.17	1.44±0.42	1.33±0.19	14.75	0.005	0.002	0.006	0.376
VEGF	1.55±0.48	1.27±0.37	1.37±0.31	0.40	0.689	0.414	0.591	0.767

Note: *P* values reflect differences in each gene expression among the three groups analyzed using one-way ANOVA.*P_1_*, *P_2_*, and *P_3_* respectively reflect the intergroup differences between the PER1-shRNA-I and Control-shRNA groups, the PER1-shRNA-I and SCC15 groups, and the Control-shRNA and SCC15 groups analyzed using the LSD test after one-way ANOVA. Values of *P<0.05* were considered significant.

### *In vivo* tumorigenesis

**Figure 5:**
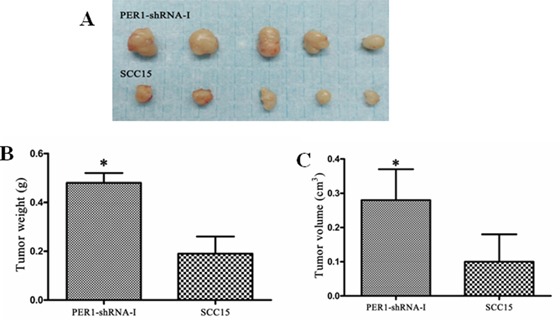
*PER1* reduces SCC15 cells' capacity for tumorigenesis *in vivo* **A.** Photographs tumors from nude mice transplanted with untreated SCC15 cells or cells expressing PER1-shRNA-I. **B and C.** Average weights and volumes of transplanted tumors from nude mice were significantly increased after *PER1* knockdown. Data were presented as the mean±SD. Student's *t*-test was used to analyze the significanceof differences. *P <0.05.

Three weeks after subcutaneous injection of untreated SCC15 cells or cells expressing PER1-shRNA-I into the backs of 10 nude mice, the tumor weights (volumes) in the PER1-shRNA-I and SCC15 groups were respectively 0.48±0.04g (0.28±0.09 cm^3^) and 0.19±0.07 g (0.10±0.08 cm^3^) (*P<0.05*) (Figure 5A-5C). This finding demonstrates that *PER1* knockdown promotes *in vivo* tumorigenesis by SCC15 cells.

## DISCUSSION

Aberrant suppression of *PER1* is strongly linked to carcinogenesis and tumor development [6, 9, 14, 15, 21]. Indeed, down-regulation of *PER1* accelerates cell growth in breast cancer, whereas up-regulation of *PER1* inhibits cell growth and promotes cell apoptosis in colon, prostate and lung cancers [6, 7, 15, 20]. Oral squamous cell carcinoma (OSCC) accounts for about 90% of oral cancers [9, 14, 19]. Our previous clinical research revealed that *PER1* expression is decreased in OSCC and closely correlated with clinical phase and lymph node metastasis in patients with OSCC [9, 19]. This implies that PER1 protein acts as an important tumor suppressor.

Clock genes sustain organismal circadian rhythm and regulate downstream CCGs to affect cellular activities [2, 5, 11-13, 18, 20, 21]. Up to now, there have been more studies focused on the molecular mechanisms by which *PER1* regulates circadian rhythm [2, 7, 14] than attempt to address the contribution of aberrant *PER1* expression to the development and progression of carcinomas. Recent studies have demonstrated that *PER1* overexpression can inhibit transcription of various downstream cell-cycle genes, including *CyclinB1, CyclinD, CyclinE, WEE-1* and *p21,* and activate *C-MYC and p53*[6, 20]. Moreover, aberrant expression of *PER1* disrupts cell cycle progression and inhibits repair of DNA damage, resulting in malignant transformation of affected cells [6, 20, 22]. However, carcinogenesis is a complex process involving cell growth, proliferation, apoptosis, invasion, metastasis and tumor angiogenesis [19, 22–24]. Recent studies have also shown that many important genes associated with tumor behavior exhibit circadian rhythm [3, 10, 22, 23, 25, 26]. These include the oncogene *MDM2* and various genes supporting cell proliferation (*KI-67*), apoptosis (*Bax*), tumor invasion and metastasis (*MMP9*) and tumor angiogenesis (*VEGF*), as well as the anti-apoptosis gene *BCL-2*. These genes thus appear to function as CCGs under the control of clock genes, though it is unclear whether they are regulated specifically by *PER1*.

Our study is the first demonstration that *PER1* knockdown in OSCC cells up-regulates expression of *KI-67, MDM2, BCL-2, MMP2* and *MMP9* and down-regulates expression of *BAX* and *TIMP-2* expression. At the same time, *PER1* knockdown enhanced tumor cell growth, proliferation, apoptosis resistance, invasion and metastasis *in vitro* and tumorigenesis *in vivo*. *TIMP-2* is an inhibitor of *MMPs* [27]. We found that *PER1* knockdown not only increased *MMP2* and *MMP9* expression, but also decreased *TIMP-2* expression, presumably leading to the observed increases in tumor cell migration and invasion. We also observed that expression of the cell cycle genes *C-MYC* and *p53* was significantly reduced after *PER1* knockdown, which is consistent with the findings of Gery et al. [20]. Moreover, *PER1* knockdown had no obvious effect on *VEGF* transcription. This may be because *VEGF* expression is not affected by *PER1*, or cell culture conditions failed to achieve hypoxia, an activator of *VEGF* transcription.

Earlier studies suggest that abnormal *PER1* expression could change circadian rhythm and/or the cell cycle, leading to carcinogenesis [6, 20, 22]. Future studies will likely clarify the specific signaling pathways affected by *PER1* as well as the contribution made by aberrant *PER1* expression to the development and progression of carcinomas. It is also anticipated that emerging from these studies will be new and effective molecular targets for the treatment of cancers.

## MATERIALS AND METHODS

### Construction of lentivirus shRNA plasmids

Based on the GenBank mRNA sequence encoding hPER1 (NM_002616.2), shRNA target points (PER1-I, CAGCACCACTAAGCGTAAATG; PER1-II, CCAGCACCACTAAGCGTAAAT; PER1-III:CCATGGACATGTCCACCTATA) were selected. Then using the design principles for RNA interference [28], three pairs of shRNA plasmids targeting *PER1*(PER1-shRNA-I, PER1-shRNA-II and PER1-shRNA-III) were designed and synthesized by Shanghai Genechem Co. Ltd (Table 2). We then separately inserted each of the shRNAs into a PLKO.1 lentiviral vector, after which the vector was linearized using AgeI and EcoRI (NEB, USA). The linearized vector fragments along with double stranded DNA fragments and vector fragments were collected and combined using T4 DNA Ligase. The scramble shRNA 5′-CCTAAGGTTAAGTCGCCCTCGCTCGAGCGAGGGCGACTTAACCTTAGG-3′ (Sigma-Aldrich, St. Louis, MO), which had no interference effects on any genes, served as the control. The recombinant vector plasmids were then transformed into freshly prepared *Escherichia coli* DH5α cells (Sangon Biotech, China). Bacterial colonies were then selected in LB medium with Amp antibiotic and cultured for 14 h at 37°C. Plasmids were extracted using a QIAGEN Plasmid Midi Kit (Qiagen, Germany), and the results of DNA sequencing were analyzed using Chromas v2.1(Technelysium, Australia).

**Table 2:** Sequences of PER1-shRNAs

	Sense strand	Antisense strand
PER1-shRNA-I	5′-CCGGCAGCACCACTAAGCGTAAATGC TCGAGCATTTACGCTTAG TGGTGCTGTTTTTG-3′	5′-AATTCAAAAACAGCACCACTAAGCGTAAA TGCTCGAGCATTTACGCTTAGTGGTGCTG-3′
PER1-shRNA-II	5′-CCGGCCAGCACCACTAAGCGTAAATC TCGAGATTTACGCTTAG TGGTGCTGGTTTTTG-3′	5′-AATTCAAAAACCAGCACCACTAAGCGTAAATC TCGAGATTTACGCT TAGTGGTGCTGG-3′
PER1-shRNA-III	5′-CCGGCCATGGACATGTCCACCTATAC TCGAGTATAGGTGGACATG TCCATGGTTTTTG-3′	5′-AATTCAAAAACCATGGACATGTCCACCTAT ACTCGAGTATAGG TGGACATGTCCATGG-3′

### Lentivirus PER1-shRNA plasmid packing

Lentiviral PER1-shRNA-I-III and scramble plasmids (8 μg) were transfected into 70-80% confluent 293T cells (Life Sciences Institute of Chongqing Medical University, China) using 20 μl of Lipofectamine 2000 (Invitrogen, USA). After incubation for 24 h, the cells were lysed and centrifuged, and four different plasmid lentiviruses were collected in the supernatant. The plasmids were then filtered through 0.45 μm cellulose acetate filters and at −80°C for later use.

### Cell transfection

SCC15 cells (Life Sciences Institute of Chongqing Medical University, China) were routinely cultured in DMEM/F12 supplemented with 10% fetal bovine serum (FBS), 100 U/mL penicillin and 100 μg/mL streptomycin in a humidified incubator at 37°C under an atmosphere of 5% CO_2_. During logarithmic growth, cells were seeded into 4 ml of medium containing 10% FBS. Thereafter 1 ml of medium containing the lentivirus vectors was added. After incubation for 24 h, stably transfected cells were selected in puromycin-containing medium (2 μg/ml), which was refreshed every other day. The transfectants was divided into 5 groups: three experimental groups expressing PER1-shRNA-I, -II or -III, a Control-shRNA group expressing scramble shRNA, and an untreated SCC15 cell group (blank control group).

### Quantitative real-time PCR assay (qRT-PCR)

qRT-PCR was performed according to the manufacturer's instructions (TaKaRa, Japan). Briefly, total RNA was extracted from cells using RNAiso Plus (Takara, Japan), after which the RNA concentration and quality was determined using a UV/Visible spectrophotometer (AmershamBiosciences, Sweden) to measure absorbance at 260 nm and 280 nm. The RNA was then reverse transcribed to cDNA synthesis using a PrimeScript RT reagent Kit according to the manufacturers (Takara, Japan) instructions. For qRT-PCR, the primers for *PER1, KI-67, MDM2, C-MYC, p53, BAX, BCL-2, MMP2, MMP9, TIMP-2* and *VEGF* were designed using Oligo17.0 software and are listed in Table 3. *β-actin* served as a normalization control. The reaction mixture for qPCR contained 12.5 μl of 2×SYBR Premix Ex TaqTMII, 2 μl of cDNA template, 1μl of 0.4 μM forward and reverse primers and double distilled H_2_O in a total volume of 25 μl. qPCR was performed using a C-1000TM Thermal Cycler (Bio-Rad, USA). The PCR protocol entailed 1 cycle at 95°C for 1.5 min and 40 cycles of 10 s at 95°C and 30s at 60°C. The cycle threshold (Ct) values were determined and normalized against the expression of β-actin in each sample, and the data were analyzed using the2^−ΔΔCt^ method. The assays were done in triplicate.

**Table 3:** Primer sequences used for quantitative real-time PCR

Gene	Forward primer sequence (5′-3′)	Reverse primer sequence (5′-3′)
PER1	CTGCTACAGGCACGTTCAAG	CTCAGGGACCAAGGCTAGTG
KI-67	TAACACCATCAGCAGGCAAA	GCAGGTCCAGTTTCTCCACT
MDM2	TCTGAAAGCACCAGCACTTG	TACTGAACACGCCTCCCATC
C-MYC	CGGAACTCTTGTGCGTAAGG	GGTTGTGAGGTTGCATTTGA
p53	TAGTGTGGTGGTGCCCTATG	CCAGTGTGATGATGGTGAGG
BAX	ATGGGCTGGACATTGGAC	GGGACATCAGTCGCTTCAGT
BCL-2	CAACACAGACCCACCCAGA	TGGCTTCATACCACAGGTTTC
MMP2	AGTTTCCATTCCGCTTCCAG	CGGTCGTAGTCCTCAGTGGT
MMP9	ACTACTGTGCCTTTGAGTCC	AGAATCGCCAGTACTTCCCA
TIMP-2	AGGCTTAGTGTTCCCTCCCT	TGTCACCAAAGCCACCTACC
VEGF	GGCAAAGTGAGTGACCTGCT	CGGTGTCTGTCTGTCTGTCC
β-actin	AGCGAGCATCCCCCAAAGTT	GGGCACGAAGGCTCATCATT

### Western blot analysis

Cells were lysed in RIPA buffer [50 mmol/L Tris-HCl (pH 7.4), 150 mmol/L NaCl, 0.5% NP-40] for 30 min at 0°C and centrifuged for 15 min (12000 rpm, 4°C). Protein expression was quantified using a BCA Protein Assay Kit according to the manufacturer's instructions (Beyotime, China). The lysates (50 mg protein) were subjected to SDS-PAGE, after which the proteins were transferred to polyvinylidene fluoride (PVDF) membranes (Millipore, USA), which were then blocked with Tris-buffered saline (TBS)-Tween containing 5% non-fat dried milk for 2 h. The membranes were then probed with rabbit polyclonal anti-hPER1 antibody (1:1000, Genetex, USA) and mouse monoclonal anti-hGAPDH antibody (1:3000, Zhongshan Golden-Bridge Biotechnology, China) overnight at 4°C, followed by horseradish peroxidase-conjugated goat anti-rabbit IgG (1:5000, Zhongshan Golden-Bridge Biotechnology, China) at 37°C for 1 h. The precipitated proteins were washed three times in PBS, and an ECL-advance Western Blot Detection System (ChemiDocXRS+, Bio-Rad, USA) was used for detection and photography. The assays were done in triplicate.

### Cell counting Kit-8(CCK-8) assay

*PER1* mRNA and protein was knocked down most efficiently in PER1-shRNA-I cells, which were used for the following experiments. PER1-shRNA-I, Control-shRNA and untreated SCC15 cells were seeded into 96 well plates to a density of 1000 cells/well and then counted every 24 h for 5 days. Medium containing 10% FBS with no cells served as an internal reference. On the day of assays, the cells were fed with 100 μl of fresh medium along with 10 μl of CCK-8 (Dojindo, Japan) solution and incubated at 37°C for 1 h. Cell growth was measured based on the absorbance at 450 nm using a microplate reader (BioTek, USA). The assays were done in triplicate to ensure the accuracy of the data.

### Colony formation assay

Approximately 100 cells collected during the logarithmic growth phase were seeded into each well of 6-well plates. The medium was replaced every other day. Cells were fixed with methanol and stained with 0.1% crystal violet after 12 days, then washed with deionized water. Colonies containing over 50 cells were counted manually (200×) under a microscope (Olympus, Japan). The colony formation rate was expressed as the percentage of colonies per numbers of inoculated cells. The experiments were repeated three times to obtain the average colony formation rate.

### Flow cytometric apoptosis assay

Cells in logarithmic growth phase were harvested by trypsinization and centrifuged for 5 min (1000 rpm, 4°C), after which the supernatant was discarded and the cell pellets were washed twice with PBS and resuspended in DMED/F12 medium at a density of 1×10^6^/ml. Aliquots of suspension (1 ml) were centrifuged for 5 min. The cell pellets were then incubated with 200 μl of AnnexinV-FITC reagent for 15 min at 22°C, and then stained with 1 ml of propidium iodide solution for 5 min at 4°C in the dark. Apoptosis was analyzed using FACSVantage flow cytometry (BD, USA). The following formula was used to calculate the tumor cells apoptotic index (AI): AI = (number of apoptotic cells/total number of cells)×100%. Each experiment was carried out in triplicate.

### Cell migration assay

Transwell chambers (Corning, USA) were divided into upper and lower chambers by an 8-μm-pore polycarbonate membrane. After addition of 1.0×10^3^ cells serum-free DMEM/F12 medium to the upper chamber and medium containing 10% FBS to the lower chamber, the chambers were incubated for 24 h at 37°C under 5% CO_2_. The medium was then aspirated from the inside of the insert, and the non-migrated cells on the upper side of the membrane were removed using a cotton swab. The membrane was fixed then with methanol for 20 min and stained with 0.1% crystal violet for 15 min. Cells that had migrated to the lower surface of the membrane were counted in 10 random microscope fields (200×) and photographed. The values reported here are the averages of triplicate experiments.

### Cell invasion assay

The experiment procedures were roughly the same as for the cell migration assay described above, except the upper surface of a polycarbonate membrane was coated with 60 μl of Matrigel (BD, USA).

### *In vivo* tumorigenesis assay

Ten specific pathogen-free (SPF) BALB/c nu/nu nude mice (female, 4-6 weeks old, 18-22 g) were purchased from the Experimental Animal Center of the Chongqing Medical University and divided into two groups: PER1-shRNA-I and SCC15. The mice in the corresponding groups were then subcutaneously injected into the right back with 0.2 ml of PBS containing 1×10^6^ PER1-shRNA-I or SCC15 cells. Three weeks later, noticeable tumors were present, and the mice were sacrificed by cervical dislocation. The tumors were immediately excised, washed with PBS, dried on filter paper and weighed using a precise balance (AA250, Denver Instrument, USA). Tumor size was measured using a caliper, and tumor volume was calculated using the formula V=0.5×L×W^2^, where V is the volume, L is the length, and W is the width. The tumors were then fixed in 4% paraformaldehyde, embedded in paraffin blocks, and cut into 4-μm slices. Routine HE staining was then performed, and the sections were observed under an optical microscope (200×). This experiment was conducted in strict accordance with the recommendations in the Guide for the Care and Use of Laboratory Animals of the Chongqing Medical University. All animal experimental protocols were approved by the Ethics Committee of Chongqing Medical University (Permit Number: CQMU 2011-28).

### Statistical analysis

All statistical analyses were performed using SPSS 17.0 software (IBM Corporation, USA). Data are expressed as the mean±SD. Comparisons between multiple groups were made using one-way analysis of variance (ANOVA). Comparisons between two groups were made using the least significant difference (LSD) test. Student's *t*-test was used to analyze differences between two groups of tumorigenesis assay. Values of p<0.05 were considered statistically significant.

## SUPPLEMENTARY FIGURE AND TABLE


