# Supplementary material for: The clock gene PER1 suppresses expression of tumor-related genes in human oral squamous cell carcinoma
Source: Oncotarget. 2016 Mar 1;7(15):20574–83. doi: 10.18632/oncotarget.7827 (PMC4991476; doi:10.18632/oncotarget.7827)
Supplement: Supplementary file 1 [file oncotarget-07-20574-s001.pdf]

SUPPLEMENTARY FIGURE AND TABLE

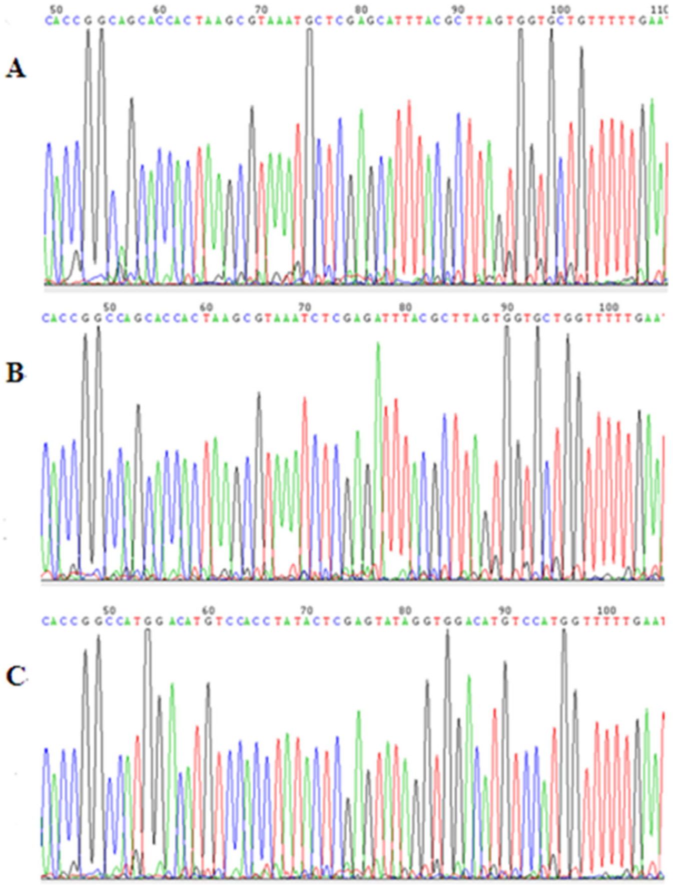

Supplementary Figure S1: DNA sequencing results of lentivirus PER1-shRNA plasmids A. PER1-shRNA-I group; B. PER1-shRNA-II group; C. PER1-shRNA-III group.

Supplementary Table S1: Sequences of PER1-shRNA interference

Group	Sense strand
PER1-shRNA-I	5’-CCGGCAGCACCACTAAGCGTAAATGCTCGAGCATTTACGCTTAGTGGTGCTGTTTTTG-3’
PER1-shRNA-II	5’-CCGGCCAGCACCACTAAGCGTAAATCTCGAGATTTACGCTTAGTGGTGCTGGTTTTTG-3’
PER1-shRNA-III	5’-CCGGCCATGGACATGTCCACCTATACTCGAGTATAGGTGGACATGTCCATGGTTTTTG-3’
